# Clostridial Mycotic Aneurysm Leading to Emphysematous Aortitis

**DOI:** 10.7759/cureus.14136

**Published:** 2021-03-27

**Authors:** Thomas G Ng, Usha Trivedi, Kajol Shah, Pierre Maldjian

**Affiliations:** 1 Internal Medicine, Rutgers University, Newark, USA; 2 Radiology, Rutgers University, Newark, USA

**Keywords:** emphysematous aortitis, clostridium, mycotic aneurysm, rutgers njms, cardiothoracic surgery

## Abstract

Mycotic aneurysms account for less than 5% of all aneurysms of the aorta, with most cases linked to infection with either *Staphylococcus* or *Salmonella* species. Emphysematous aortitis is a rare consequence of mycotic aneurysms and is associated with high morbidity and mortality. It typically occurs from infection superimposed on already damaged endothelium, which is commonly seen in conditions such as atherosclerosis. This report discusses the presentation and relevant imaging findings of a unique case of emphysematous aortitis from Clostridial infection of the thoracic aorta. The patient was a 66-year-old male with a past medical history of end-stage renal disease, arteriovenous fistula for dialysis, hypertension, and diabetes, who presented with tachycardia and tachypnea. Computed tomography of the chest showed inflammatory changes of the thoracic aorta with gas bubbles along the aortic wall, and post-mortem aortic tissue cultures were positive for *Clostridium innocuum*. Although emphysematous aortitis is rare, the radiographic findings are strikingly characteristic and should prompt immediate and aggressive management.

## Introduction

Emphysematous aortitis is a rare consequence of underlying mycotic aneurysm, an uncommon cause of arterial dilation resulting from infection of damaged vessel endothelium. A misnomer, mycotic aneurysm is usually caused by bacterial rather than fungal infection, and accounts for 0.7%-1.3% of all surgically treated aneurysms. Common risk factors include immunosuppression, intravenous drug use, iatrogenic vessel trauma, atherosclerosis, and endocarditis. This phenomenon can not only affect the thoracic and abdominal aorta but also the intracranial and peripheral arteries. Most cases have been linked to infection with either *Staphylococcus* or *Salmonella* species [1]. This case reports Clostridial aortic infection, an uncommon cause of mycotic aneurysm, leading to arterial myonecrosis and subsequent aortitis diagnosed on computed tomography (CT) imaging.

## Case presentation

**Figure 1:**
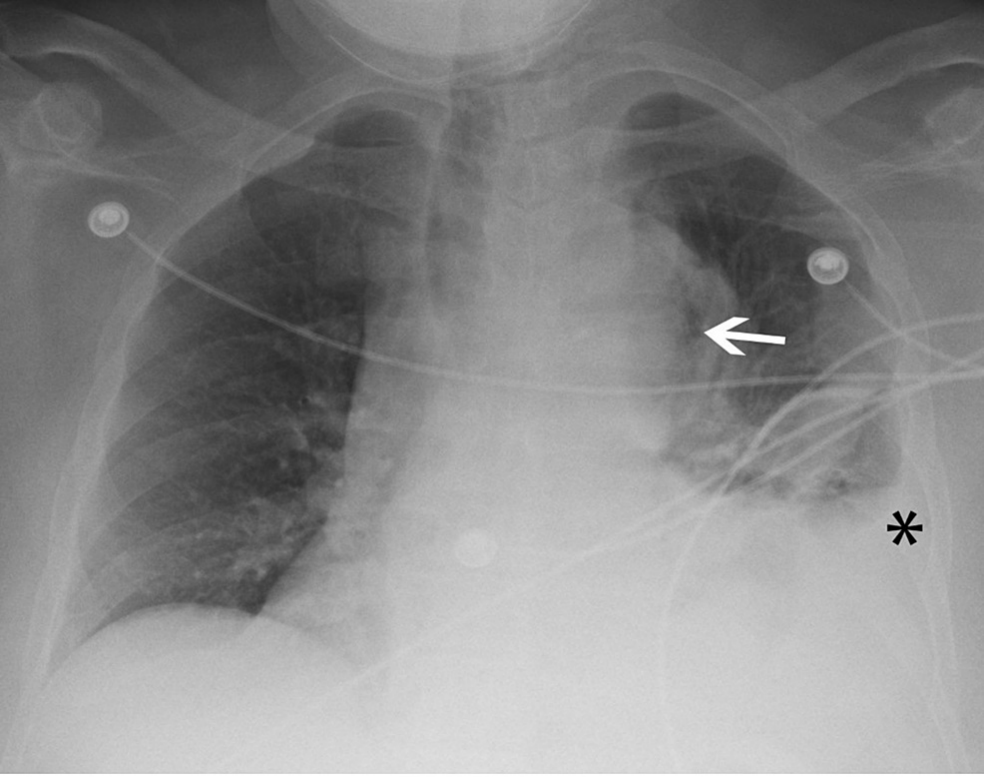
Chest radiograph shows widening of the mediastinum with a lucent region (arrow) along the left border of the thoracic aorta. Note left pleural effusion (*).

**Figure 2:**
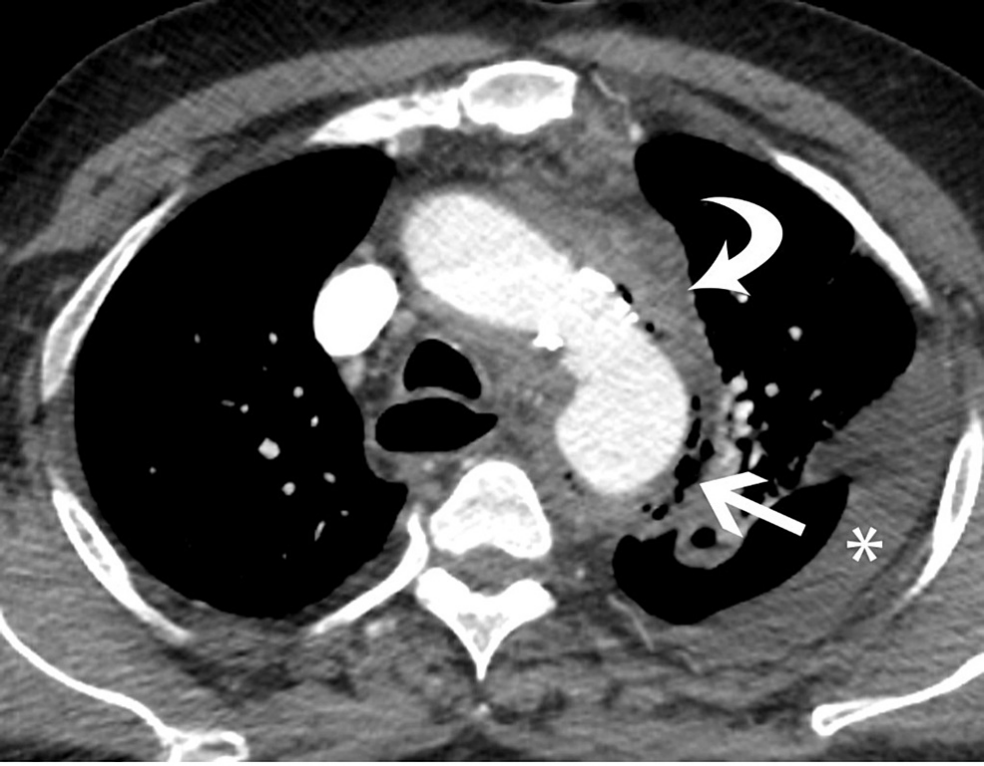
Axial image from CT scan of the chest shows fluid and inflammatory changes adjacent to the aortic arch (curved arrow), gas bubbles along the aortic wall (straight arrow) and left pleural effusion (*).

**Figure 3:**
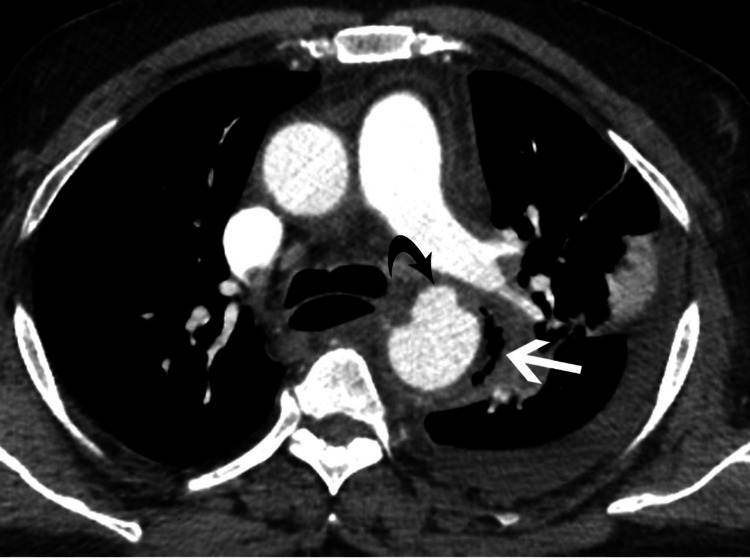
Axial image at a slightly lower level shows an aneurysm (curved black arrow) projecting from the anterior wall of the proximal descending aorta with gas bubbles (white arrow) along the left lateral wall.

**Figure 4:**
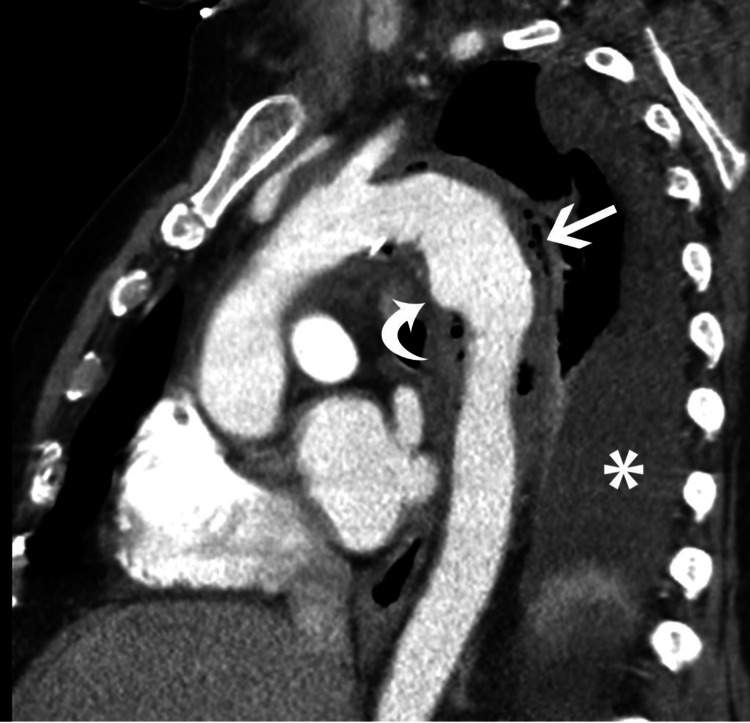
Oblique sagittal reformatted image in the plane of the aortic arch shows fluid and inflammatory changes along with gas bubbles (straight white arrow) along the aortic wall and aneurysm (curved white arrow) projecting anteriorly from the proximal descending thoracic aorta. Note left pleural effusion (*).

The patient was a 66-year-old man with a past medical history of end-stage renal disease with an arteriovenous fistula placed five years prior in the left arm for dialysis, hypertension, and diabetes who presented with one-week history of non-bloody diarrhea, vomiting, and fever. On physical examination, the patient was tachycardic and tachypneic. The patient’s initial laboratory results were significant for a leukocytosis of 23,000/µL with elevated inflammatory markers including a D-dimer of 4,700 ng/mL, a C-reactive protein of 382 mg/L, and a procalcitonin of 25.26 ng/mL. An initial chest radiograph was significant for a widened mediastinum with a lucent region alongside the thoracic aorta (Figure 1). Computed tomography showed a 1.4 cm aneurysmal out-pouching of the proximal descending thoracic aorta along with inflammatory changes and gas bubbles in the periaortic soft tissues extending from the aortic arch to the mid descending thoracic aorta diagnostic of emphysematous aortitis (Figures 2-4). There was also a large, loculated, left-sided pleural effusion with atelectasis of the left lower lobe initially concerning for empyema, but likely reactive as thoracentesis revealed polymorphonuclear lymphocytes with negative bacterial cultures.

Blood cultures revealed *Clostridium subterminale* bacteremia. The patient was worked up to determine the primary source of bacterial infection. Since Clostridial infection can be associated with pathology in the gastrointestinal tract, the patient underwent colonoscopy and esophagogastroduodenoscopy, which were both negative. Abdominal CT also showed no evidence of gastrointestinal lesion or malignancy. Echocardiography, performed to exclude seeding from endocarditis, was also negative. The patient urgently underwent ascending aorta and distal arch reconstruction with a left carotid-subclavian bypass. Unfortunately, despite multiple revisions of the aortic graft, broad-spectrum antibiotics, and vasopressor support, the patient succumbed to cardiac arrest from multi-organ failure due to septic shock. Post-mortem aortic tissue cultures were positive for *C. innocuum*.

## Discussion

Emphysematous aortitis is a rare but often fatal sequelae of mycotic aneurysm. Infection of the aorta can occur as a complication of surgery or interventional procedure, bacteremic seeding from a source in the gastrointestinal tract or endocarditis, or spread of infection from a source contiguous to the aorta, such as lung or vertebra. Risk factors include intravenous drug use, history of recent vascular procedure, bacteremia, immunosuppression, atherosclerosis, and pre-existing aneurysms. Most cases are usually caused by *Staphylococcus* or *Salmonella* species, reported in 71% and 24% of cases, respectively. *Clostridium* species comprise approximately 0.7% of cases.

As *Clostridia* are gas-producing organisms, emphysematous changes are present in 92.6% of Clostridial mycotic aneurysms. Spore-forming obligate anaerobes, *Clostridia* can proliferate in necrotic or anoxic areas of injury such as the damaged endothelial tissue of vessels. The release of Clostridial toxins and enzymes, such as hyaluronidase and hemolysin, produces gas gangrene infection. Curiously, the patient was found to have bacteremia with two different *Clostridium* species, *C. subterminale* on blood cultures and *C. innocuum* in the aortic wall. *Clostridium* bacteria are readily found in soil and in the gastrointestinal flora, and reported cases of *C. subterminale* bacteremia have been associated with gastrointestinal mucosal damage and colorectal malignancy. In rare cases, diverticulitis and ileus have been implicated as causes for *Clostridium* emphysematous aortitis. The presence of *C. innoccum* infection is particularly notable, as most *Clostridium* infections in the aortic wall are caused by *C. perfringens* or *C. septicum* [2]. *Clostridium innoccuum* has been associated with severe acquired immunodeficiency syndrome (AIDS), leukemia, or underlying malignancy, none of which our patient had. Our patient’s negative gastrointestinal workup suggests a different source of infection for both species. Diabetes mellitus has been associated with gas gangrene infections in patients without preceding trauma and was likely a risk factor for our patient. We speculate that contamination of the patient’s arteriovenous fistula may have resulted in bacteremic seeding of the bloodstream of the aorta during the hemodialysis.

The diagnosis of emphysematous aortitis involves a combination of findings. The typical presentation of this disease is characterized by pain, fever, and signs of infection, along with history of relevant risk factors including arterial disease or an immunocompromised state [3]. Of those diagnosed, 77.3% of patients presented with pain, most commonly abdominal and back pain, 45.5% presented with fevers, and 9.1% had gastrointestinal symptoms such as nausea, vomiting, and diarrhea, as in our case [4]. Laboratory findings that support the diagnosis include elevated inflammatory markers, presence of bacteremia, and positive aneurysm tissue cultures from the affected blood vessel.

Findings on CT imaging suggestive of an infected thoracic aneurysm include the presence of a saccular or multilobed aneurysm with inflammatory changes in the mediastinal fat adjacent to the aorta and perivascular fluid. Air along the aortic wall is diagnostic of emphysematous aortitis from gas-forming bacterial infection. When recognized, this requires urgent and aggressive management.

Treatment of mycotic aneurysms involves prompt resection and debridement of infected tissues and long-term antibiotic therapy. Patients with mycotic aneurysms also require antibiotic coverage for *Staphylococcus* and *Salmonella *infections. Duration of treatment can last up to six weeks or more depending on the pathogenic organism, patient comorbidities, and severity of disease. However, despite aggressive treatment, many patients suffer poor outcomes. The overall hospital mortality of patients with mycotic aortic aneurysms was 23%-31% with 7.7% mortality within the first 30 days after surgery. Causes of death include aneurysm rupture, progression of mediastinitis, graft infection, pneumonia, or multi-organ failure from septic shock [5].

## Conclusions

Emphysematous aortitis is a rare consequence of mycotic aneurysms and is associated with high morbidity and mortality. Diagnosis can be presumed when imaging and laboratory findings are consistent with acute infection and confirmed by bacterial culture. Treatment involves both aggressive surgical reconstruction of the damaged vessel as well as long-term intravenous antibiotic therapy. The presence of air in the aortic wall with surrounding inflammatory changes on CT should prompt urgent and aggressive management.
